# High Spatial Resolution Infrared Micro-Spectroscopy Reveals the Mechanism of Leaf Lignin Decomposition by Aquatic Fungi

**DOI:** 10.1371/journal.pone.0060857

**Published:** 2013-04-05

**Authors:** Janice L. Kerr, Darren S. Baldwin, Mark J. Tobin, Ljiljana Puskar, Peter Kappen, Gavin N. Rees, Ewen Silvester

**Affiliations:** 1 Department of Environmental Management and Ecology, La Trobe University, Wodonga, Victoria, Australia; 2 Murray Darling Freshwater Research Centre (MDFRC) and CSIRO Land and Water, La Trobe University, Wodonga, Victoria, Australia; 3 The Australian Synchrotron, Clayton, Victoria, Australia; 4 Centre for Materials and Surface Science and Department of Physics, La Trobe University, Bundoora, Victoria, Australia; Dowling College, United States of America

## Abstract

Organic carbon is a critical component of aquatic systems, providing energy storage and transfer between organisms. Fungi are a major decomposer group in the aquatic carbon cycle, and are one of few groups thought to be capable of breaking down woody (lignified) tissue. In this work we have used high spatial resolution (synchrotron light source) infrared micro-spectroscopy to study the interaction between aquatic fungi and lignified leaf vein material (xylem) from River Redgum trees (*E. camaldulensis*) endemic to the lowland rivers of South-Eastern Australia. The work provides spatially explicit evidence that fungal colonisation of leaf litter involves the oxidative breakdown of lignin immediately adjacent to the fungal tissue and depletion of the lignin-bound cellulose. Cellulose depletion occurs over relatively short length scales (5–15 µm) and highlights the likely importance of mechanical breakdown in accessing the carbohydrate content of this resource. Low bioavailability compounds (oxidized lignin and polyphenols of plant origin) remain in colonised leaves, even after fungal activity diminishes, and suggests a possible pathway for the sequestration of carbon in wetlands. The work shows that fungi likely have a critical role in the partitioning of lignified material into a biodegradable fraction that can re-enter the aquatic carbon cycle, and a recalcitrant fraction that enters long-term storage in sediments or contribute to the formation of dissolved organic carbon in the water column.

## Introduction

In many aquatic ecosystems (rivers, lakes and wetlands) leaf litter is an important source of carbon, contributing to both the aquatic food web as well as the formation of stored (recalcitrant) organic carbon [1]. The formation and stability of stored carbon reserves is critical to global carbon cycles and future climate trajectories [2]. Wetlands are particularly important with respect to carbon storage; wetlands occupy somewhere between 2 and 6% of the world's land surface area [3], but contain about one third of the organic matter stored in the world's soils [4]. There is however still debate on whether wetlands are a significant global source or sink for carbon [3]. The formation of new stored carbon in wetlands occurs through the action of micro-organisms, converting plant materials into new recalcitrant compounds [5]. Fungi are likely important in the decomposition of vascular plant leaf litter as they are thought to be one few organism groups able to access the carbohydrate content of lignified tissue [6], [7]. Studies using simplified lignin-like substrates have shown that fungi use depolymerisation exo-enzymes such as ligninases to cleave carbon-carbon and carbon-oxygen bonds [8], [9], [10] although there is very little direct evidence for this process in natural systems.

While significant information about the fungal decomposition of plant material can be obtained from bulk analysis techniques (fungal biomass [11], induced sporulation [11], [12], leaf composition [13], [14]) and DNA-based measurements of community structure [15], [16], such techniques do not provide spatially explicit data about the interaction of fungi with plant substrates. Infrared (IR) micro-spectroscopy is ideally suited to the study of bio-molecular changes in leaf tissue during fungal leaf decomposition; IR spectra allow the discrimination of leaf tissue composition [17], and leaf anatomy is reproducible between samples so changes in spatial distributions are readily recognised. The coupling of IR micro-spectroscopy with synchrotron light sources offers considerable advantages in terms of spatial resolution, allowing investigation of the interface between fungal tissue and leaf substrate at relevant length scales [17]. In particular, IR micro-spectroscopy allows direct examination of changes in leaf lignin composition is close proximity to fungal tissue, and therefore the opportunity to verify the depolymerisation of the lignified substrate.

In Australian lowland wetlands the leaf litter from *E. camaldulensis* (the River Redgum) is a major source of non-aquatic carbon [18]. Leaf fall predominantly occurs during the austral summer, with leaves accumulating on the floodplain before entering aquatic ecosystems during a flood [19]. Here we have used focal plane array Fourier transform infrared spectroscopy (FPA-FTIR) and synchrotron light source Fourier transform infrared spectroscopy (S-FTIR) microscopic techniques to map the chemical composition of *Eucalyptus camaldulensis* leaves through the terrestrial and aquatic decomposition processes, focusing on changes in the leaf mid-vein anatomy. Leaf samples were selected at the stages of the decomposition process where maximum contrast was expected: a fresh *E. camaldulensis* leaf prior to any apparent decomposition, a leaf terrestrially aged on a floodplain, and leaves that were first terrestrially aged and then conditioned in a wetland for 15 and 107 days.

## Results and Discussion

### Leaf anatomy and histology

**Figure 1:**
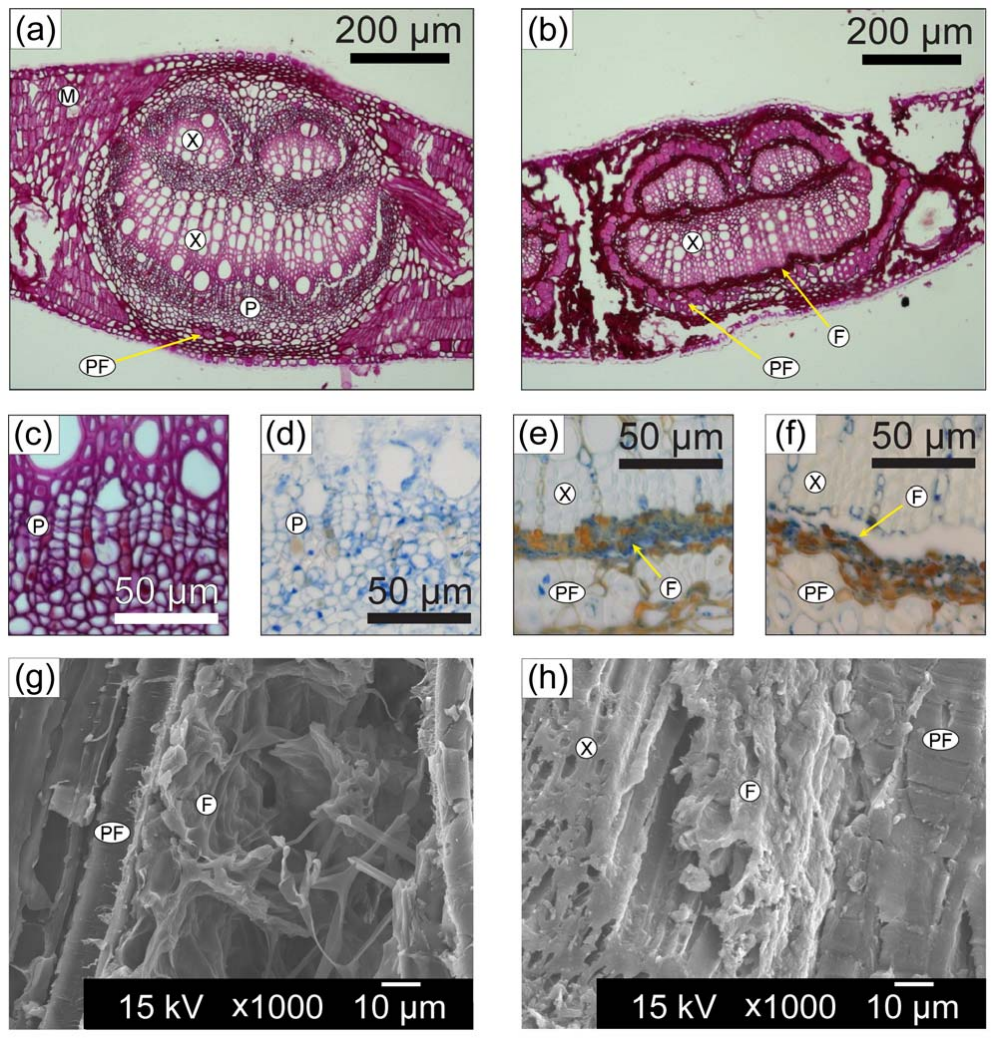
Histological and SEM images of the mid-vein of fresh, terrestrially aged and aquatically conditioned *E. camaldulensis* leaves. (**a**) Periodic Acid-Schiff (PAS) stained fresh leaf. (**b**) PAS stained leaf aquatically conditioned for 15 days. (**c**) PAS stained fresh leaf (phloem). (**d**) Lacto-phenol cotton blue (LPCB) stained fresh leaf (phloem). (**e**) LPCB stained terrestrially aged leaf (xylem-phloem fibre interface). (**f**) LPCB stained 15 day aquatically conditioned leaf (xylem-phloem fibre interface). (**g**) SEM image of fungal hyphae around phloem fibres for a terrestrially aged leaf. (**h**) SEM image of fungal-containing material between xylem and phloem fibres for 15 day aquatically conditioned leaf. X = xylem; P = phloem; PF = phloem fibres; F = fungal tissue; M = mesophyll.

Histological sections of fresh *E. camaldulensis* leaves are shown in Figure 1 stained with periodic acid-Schiff's (PAS) (Figure 1(a) and (c)) and lacto-phenol cotton blue (LPCB) (Figure 1(d)). Terrestrial aging combined with leaching and colonisation by aquatic micro-organisms (conditioning) results in the removal of readily biodegradable tissue, including the mesophyll, phloem and collenchyma in the mid-vein region (15 days aquatic conditioning; Figure 1(b)). When compared to the fresh leaf, LPCB stain indicates the presence of fungal tissue in both terrestrial (Figure 1(e)) and aquatically conditioned leaves (Figure 1(f)), located between the xylem and phloem fibres, as well as between phloem fibres and cuticle, and extending into the mesophyll space (later not shown). The presence of fungal tissue in the terrestrially aged leaf is confirmed by Scanning Electron Microscopy (SEM), both outside the phloem fibres (Figure 1(g)) as well as adjacent to the xylem (see Supplementary Figure S1). For the aquatically aged leaf the material identified by LPCB stain as containing fungi is more amorphous (Figure 1(h)), consistent with lower apparent abundance of fungi in this state, although individual fungal structures can be identified (see Supplementary Figure S2).

### Infrared micro-spectroscopy

**Figure 2:**
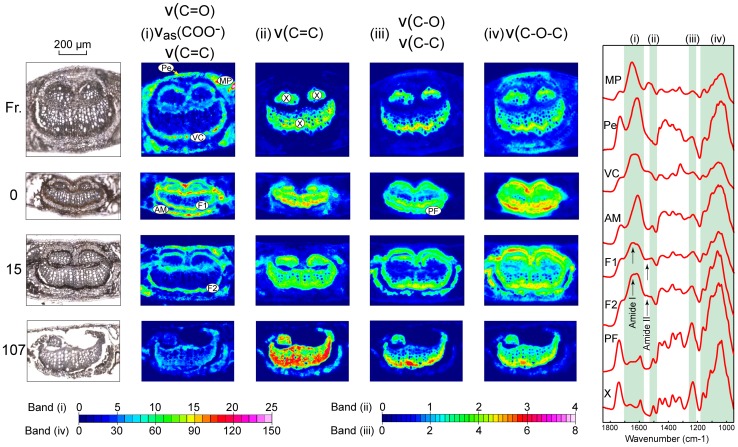
Bright field photomicrographs and FPA-FTIR transmission images of the mid-vein of *E. camaldulensis* leaves. Shown are: a fresh leaf collected from a living tree (**Fr.**), a terrestrially aged leaf collected from floodplain litter (**0**) and terrestrially aged leaves that were subsequently submerged in a floodplain wetland for 15 (**15**) and 107 (**107**) days. IR maps are shown for wavenumber regions corresponding to: (**i**) stretching modes of carbonyl, aromatic and carboxylate groups (1705-1570 cm^−1^); (**ii**) stretching modes of aromatic groups (1530-1480 cm^−1^); (**iii**) C-C and C-O stretching modes (particularly of lignin) (1260-1210 cm^−1^); and (**iv**) C-O-C stretching modes of carbohydrates (1180-950 cm^−1^). Single pixel point spectra (raw data extracted from FPA maps) of selected leaf and fungal tissue types are shown on the right; shaded areas on these spectra are the integration ranges for wavenumber regions (**i**)–(**iv**). MP = mesophyll protein, Pe = pectin, AM = aromatic material, F = fungal-containing material All other labels as for Fig. 1. Refer to Tables 1 and 2 for band assignments.

**Figure 3:**
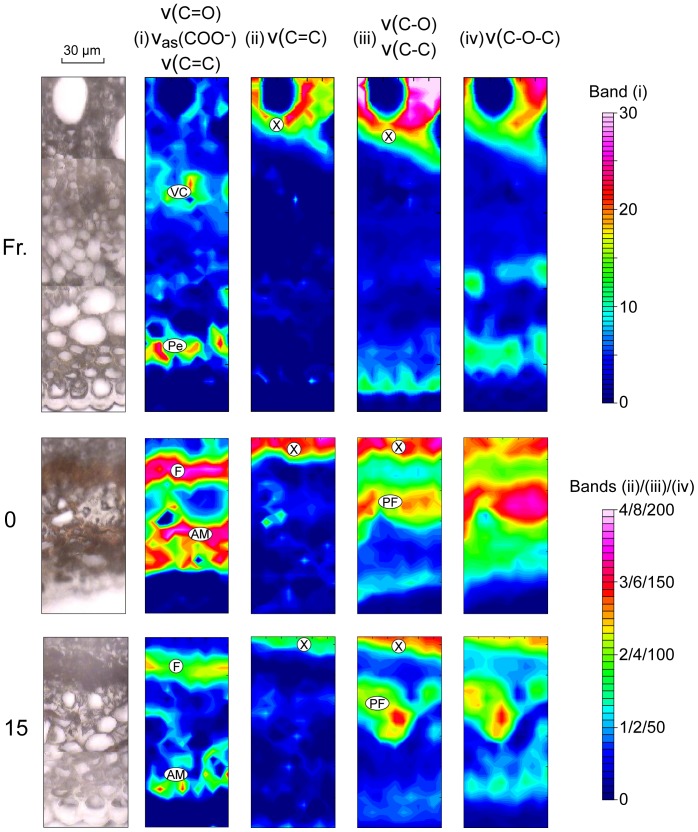
Bright field photomicrographs and transmission synchrotron IR maps for sections of *E. camaldulensis* mid-vein. from xylem to cuticle (same samples as in Fig. 2). Shown are: Fresh leaf (**Fr.**), terrestrially aged leaf (**0**), and 15 days aquatic decomposed leaf (**15**). IR wavenumber regions and tissue identifiers same as Fig. 2. Refer to Tables 1 and 2 for band assignments.

Figure 2 shows FPA-FTIR maps for a fresh leaf (**Fr.**), terrestrially aged leaf (**0**), a leaf conditioned in a wetland for 15 days (**15**) and a leaf conditioned for 107 (**107**) days. The characteristic IR active bands of proteins and chitin (observed in fungal hyphae [20]) are amide I (ν(C = O); 1660-1640 cm^−1^), amide II (ν(C-H) & ν(N-H); 1550-1535 cm^−1^) and amide III (ν(C-H) & δ(N-H); ∼1320 cm^−1^) [21], [22]. We have mapped the protein-chitin distribution using a wavenumber range that includes amide I (Figure 2 (i); 1705-1570 cm^−1^). In the fresh leaf, the carboxylate band of pectin (ν_as_(COO^−^); 1616 cm^−1^) also absorbs in this range, so this wavenumber range does not uniquely map these biomolecule types. Unambiguous protein regions in the fresh leaf mid-vein occur in the mesophyll proximate to the vascular bundle, and between the xylem and phloem, identified as vascular cambium. The remaining regions of the fresh leaf sample that map with high intensity in this wavenumber range are identified from point spectra as containing pectin. Higher resolution S-FTIR maps of a section of Fresh leaf (xylem to cuticle; Figure 3 (i)) show vascular cambium protein as isolated regions between the xylem and phloem, and the region of high pectin coinciding with collenchyma cells.

The terrestrially aged leaf (**0**) shows a very high intensity in wavenumber range (i), localised around the lignified tissues, and identified previously as containing fungal tissue through LPCB staining (Figure 1(e) & Figure 2 (i)). Point spectra of the region containing fungi indicate a mixture of protein-chitin and aromatic compounds, with wavenumber range (i) co-mapping Amide I and the aromatic quadrant ring stretch [21]. Fungal melanins are also reported to have a strong IR absorbance in this region due to both the aromatic quadrant ring stretch and antisymmetric stretch of carboxylate groups (ν_as_(COO^−^)) [23]. We are unable to definitively identify this material, although based on bright-field colour, consider it likely to be either eucalypt tannins (polyphenols) or fungal melanin, and is referred to here as ‘aromatic material (AM)’. Areas with relatively higher amounts of AM are found between the phloem fibres and cuticle, while areas richer in protein-chitin (labelled as ‘fungi’ (F)) occur between the xylem and phloem fibres (Figure 2 (i) & Figure 3 (i)). The leaf sample corresponding to 15 days aquatic conditioning (**15**) has a similar distribution and composition (protein-chitin and AM) of fungal-containing material, although diminished in intensity, particularly between the phloem fibres and cuticle. For the leaf collected after 107 days (**107**) the intensity in this wavenumber range is weak across the sample.

Lignified material in leaf sections has been mapped using two distinctive lignin bands – the aromatic ν(C = C) (semicircle ring stretch; 1530-1480 cm^−1^; Figure 2 & Figure 3 – wavenumber range (ii)) and the broader combined ν(C-C) & ν(C-O) bands (1260-1210 cm^−1^; Figure 2 & Figure 3 – wavenumber range (iii)). Wavenumber range (ii) strongly maps xylem tissue in all samples whereas range (iii) maps both xylem and phloem fibres, suggesting that phloem fibres have less aromatic character than lignin. Phloem fibres, are less developed in the fresh leaf sample (**Fr.**), but clearly evident in the terrestrially aged (**0**) and 15 day aquatic conditioned (**15**) samples.

Carbohydrates are mapped using the characteristic broad envelope in the wavenumber range 1180-950 cm^−1^ corresponding to ν(C-O-C) bands (Figure 2 & Figure 3 – wavenumber range (iv)). Carbohydrates, while clearly distributed across the entire sample in the fresh leaf, are highest in the lignified material (xylem and phloem fibres) and in the collenchyma, in the later case correlated with regions of high pectin (Figure 2 (i); Figure 3(i)). In the terrestrially aged leaf (**0**) and aquatic conditioned leaves (**15** & **107**), regions of high carbohydrate content are restricted to xylem and phloem fibres. S-FTIR maps show that in the terrestrially aged leaf (**0**) and 15 day aquatic conditioned leaf (**15**), fungal-containing material tissue occupies the region between the xylem and phloem fibres containing high levels of carbohydrate (Figure 3 (i) & (iv)).

### Changes in xylem composition at the fungal interface

The S-FTIR spectroscopic data acquired for terrestrially aged and aquatic conditioned leaf samples have been used to generate line scan plots across the fungal tissue-xylem interface. Pixel blocks, encompassing both fungal-containing tissue and xylem, from terrestrially aged, 15 days aquatic conditioning and 107 days aquatic conditioning were co-added and analysed by multivariate curve resolution (MCR) techniques (the selected pixel blocks for **0**, **15** and **107** are shown in supplementary information; Figure S3, Figure S4 and Figure S5). These data are at pixel resolution (5 µm) and have been aligned at the tissue boundary, allowing the line scan plots to be generated at 5 µm steps from the interface. Two wavenumber ranges were analysed: (i) ‘carbonyl region’ 1800 cm^−1^–1480 cm^−1^; including: carbonyl groups (ester, ketone, aldehyde, Amide I), Amide II , carboxylate groups and aromatic groups, and (ii) ‘carbohydrate region’ 1190 cm^−1^–850 cm^−1^; including all carbohydrate ν(C-O-C) bands (see Tables 1 and 2).

**Table 1:** Experimentally determined IR bands in the region 1800–950 cm^−1^ for leaf tissue bio-molecule groups (ν – stretch; ν_as_ – asymmetric stretch; δ – bend).

Bio-molecule	Wavenumber (cm^−1^)	Band assignment	Notes	Ref.
Lignin	1740	ν(C = O)	Ester carbonyl	[17]
	1665	ν(C = O)	Conjugated ketone or aldehyde	[24], [25]
	1592	ν(C = C)	Aromatic (quadrant ring stretch)	[17]
	1503	ν(C = C)	Aromatic (semicircle ring stretch)	[17]
	1230	ν(C-C), ν(C-O)		[17]
Protein/Chitin	1650	ν(C = O)	Amide I	[17], [34]
	1535	ν(C-H) & ν(N-H)	Amide II	[17], [34]
	1322	ν(C-H) & δ(N-H)	Amide III	[17], [34]
Pectin	1732	ν(C = O)	Ester carbonyl	[17]
	1616	ν_as_(COO^-^)		[17]
Tannin	1727	ν(C = O)	Ester carbonyl	[21], [35]
	1612	ν(C = C) & ν(C = O)	Aromatic and aromatic conjugated C = O	[21], [35]
	1513	ν(C = C)	Aromatic	[21], [35]
Carbohydrate	1190-970	ν(C-O-C)		[17], [27]

**Table 2:** Wavenumber range limits and corresponding absorption bands for standardised integration method applied to all FPA and synchrotron IR maps (Figures 2 and 3).

Integration region no.	Wavenumber range (cm^−1^)	Mapped bands
(i)	1705-1570	ν(C = O); Amide I
		ν_as_(COO^−^)
		ν(C = C); aromatic quadrant ring stretch
(ii)	1530-1480	ν(C = C); aromatic semicircle ring stretch
(iii)	1260-1210	ν(C-C), ν(C-O) (lignin)
(iv)	1180-950	ν(C-O-C) (carbohydrates)

#### Multivariate curve resolution (MCR) analysis - carbonyl region

**Figure 4:**
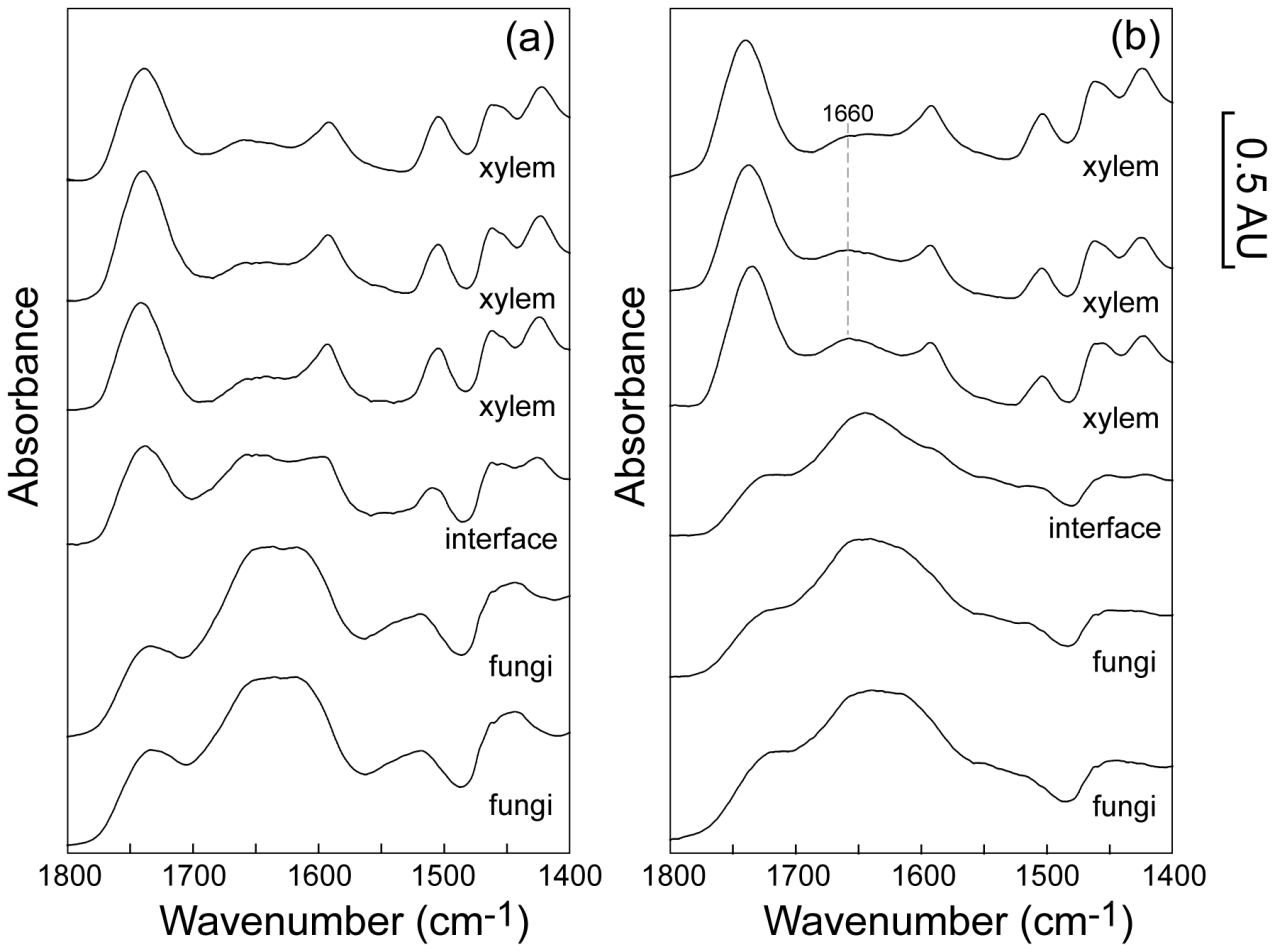
Example single pixel line scan S-FTIR spectra for the ‘carbonyl’ region across the fungal tissue-xylem interface. (wavenumber range: 1800 cm^−1^–1480 cm^−1^) for: (**a**) a terrestrially aged *E. camaldulensis* leaf (**0**) and (**b**) an *E. camaldulensis* leaf after 15 days aquatic decomposition (**15**). Note: differences in spectra recorded for fungal-containing material in **0** and **15** likely reflect differing relative amounts of protein/chitin and aromatic materials in these samples.

An example of the spectral changes that occur across the fungal tissue-xylem interface in the wavenumber range 1800 cm^−1^–1480 cm^−1^ is shown in Figure 4: part (a) shows single pixel S-FTIR spectra for the terrestrially aged leaf (**0**) and part (b) shows single pixel S-FTIR spectra after 15 days aquatic decomposition (**15**). The xylem spectra for **15** are distinctly different to that for **0** immediately adjacent to the interface between the two tissue types. In particular, for **15** the peak at ∼1660 cm^−1^ (attributed to conjugated aldehyde or ketone groups of lignin [24], [25]) increases towards the interface, likely due to a real increase in the concentration of these groups.

**Figure 5:**
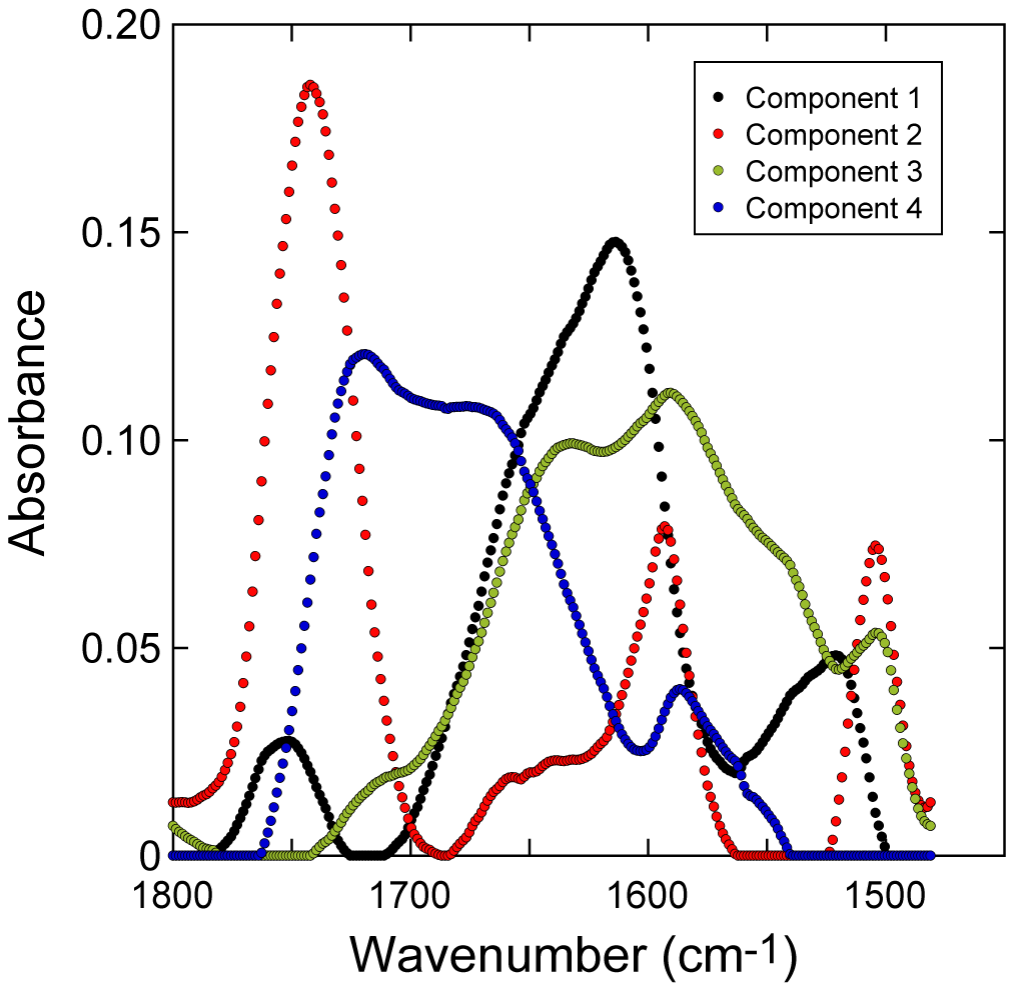
Component spectra for the ‘carbonyl’ region. (wavenumber range: 1800 cm^−1^–1480 cm^−1^) obtained from multivariate curve resolution (MCR) analysis of multiple pixel blocks (**0**, **15** & **107** – co-added) across the fungal tissue-xylem boundary. Spectral features of components described in main text.

A four component MCR fit provides a good description of experimental spectra in the 1800 cm^−1^–1480 cm^−1^ wavenumber range for the co-added pixel blocks; additional components did not significantly improve the goodness-of-fit parameter (see Experimental Methods). The MCR component spectra (Figure 5) can be understood as follows. Component 1 has bands corresponding to aromatic material (AM) with the aromatic quadrant ring stretch (1613 cm^−1^) and semi-circle ring stretch (1520 cm^−1^) as well as a weaker ester carbonyl band (1750 cm^−1^). Component 2 is very similar to that expected for pure (unoxidized) lignin and includes ester carbonyl (1740 cm^−1^), conjugated aldehyde or ketone (1660 cm^−1^) and aromatic bands (1595 cm^−1^ and 1505 cm^−1^). Component 3 includes features associated with AM (aromatic bands at 1590 cm^−1^ and 1505 cm^−1^) as well as both Amide I (1635 cm^−1^) and Amide II (1540 cm^−1^). Component 4 includes a low energy ester carbonyl or unconjugated ketone (1720 cm^−1^) plus conjugated aldehyde/ketone (∼1670 cm^−1^), and within the xylem is interpreted here as representing features of oxidized lignin. The region containing fungal tissue is fitted as combinations of components 1 and 3, with smaller amounts of component 4, allowing for variable quantities of protein/chitin and aromatic materials; component 4 is required (in part) to fit the ester carbonyl feature associated with fungal and AM (see Figure 4). The spectra in the xylem are fitted predominantly as combinations of components 2 & 4.

For each pixel block we have determined the average fractional contribution of the four spectral components across the fungal-xylem interface, after alignment at the xylem-fungi interface (defined as the zero position). Position alignment was based on the bright field image for each sample. This positioning is justified *a posteriori* from line scans for fungal tissue (components 1 & 3) and lignin (component 2) across the fungal tissue-xylem boundary for **0**, **15** and **107** (See supplementary information; Figure S3, Figure S4 and Figure S5).

**Figure 6:**
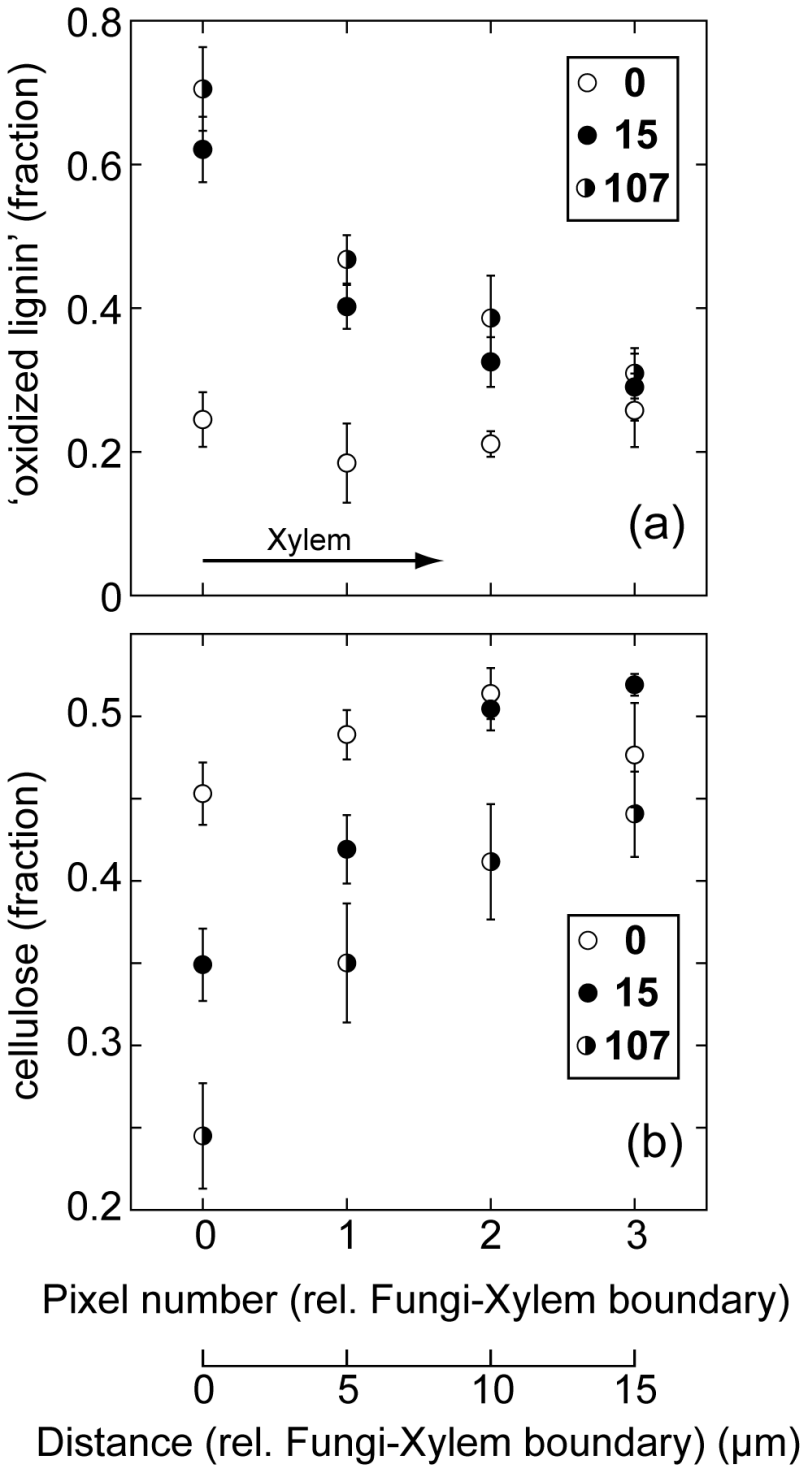
Line scan data in mid-vein xylem. for a terrestrially aged *E. camaldulensis* leaf (**0**), a leaf after 15 days decomposition in a wetland (**15**) and a leaf after 107 days decomposition (**107**), all aligned at the interface between xylem and fungal-containing material (zero position). Shown are: (**a**) average fractional contributions of component 4 (‘oxidized lignin’) to experimental spectra; (**b**) average fractional contributions of cellulose bands to the carbohydrate absorption envelope. Error bars are 2×standard error.

Line scans for the fractional contributions of component 4 (‘oxidized lignin’) within the xylem are shown in Figure 6(a) for **0**, **15** and **107**. In both **15** and **107** the concentrations of this component increase towards the interface. Given that experimental spectra within the xylem approximate to a two component fit of oxidized lignin (component 4) and unoxidized lignin (component 2), in these same samples the concentrations of unoxidized lignin decrease towards the fungal boundary (See: Figures S3, S4, S5). As noted above component 4 has a strong absorption band at ∼1670 cm^−1^, attributed to conjugated aldehyde or ketone groups [24], [25]. Oxidative depolymerisation by lignin peroxidase (ligninase) is expected to produce aromatic aldehydes and ketones [10], [26]. The spectroscopic evidence for increased abundance of these functional groups in the xylem immediately adjacent to fungal material for **15** and **107** is consistent with lignin depolymerisation through exo-enzyme ligninase activity.

#### Multivariate curve resolution (MCR) analysis - carbohydrate region

**Figure 7:**
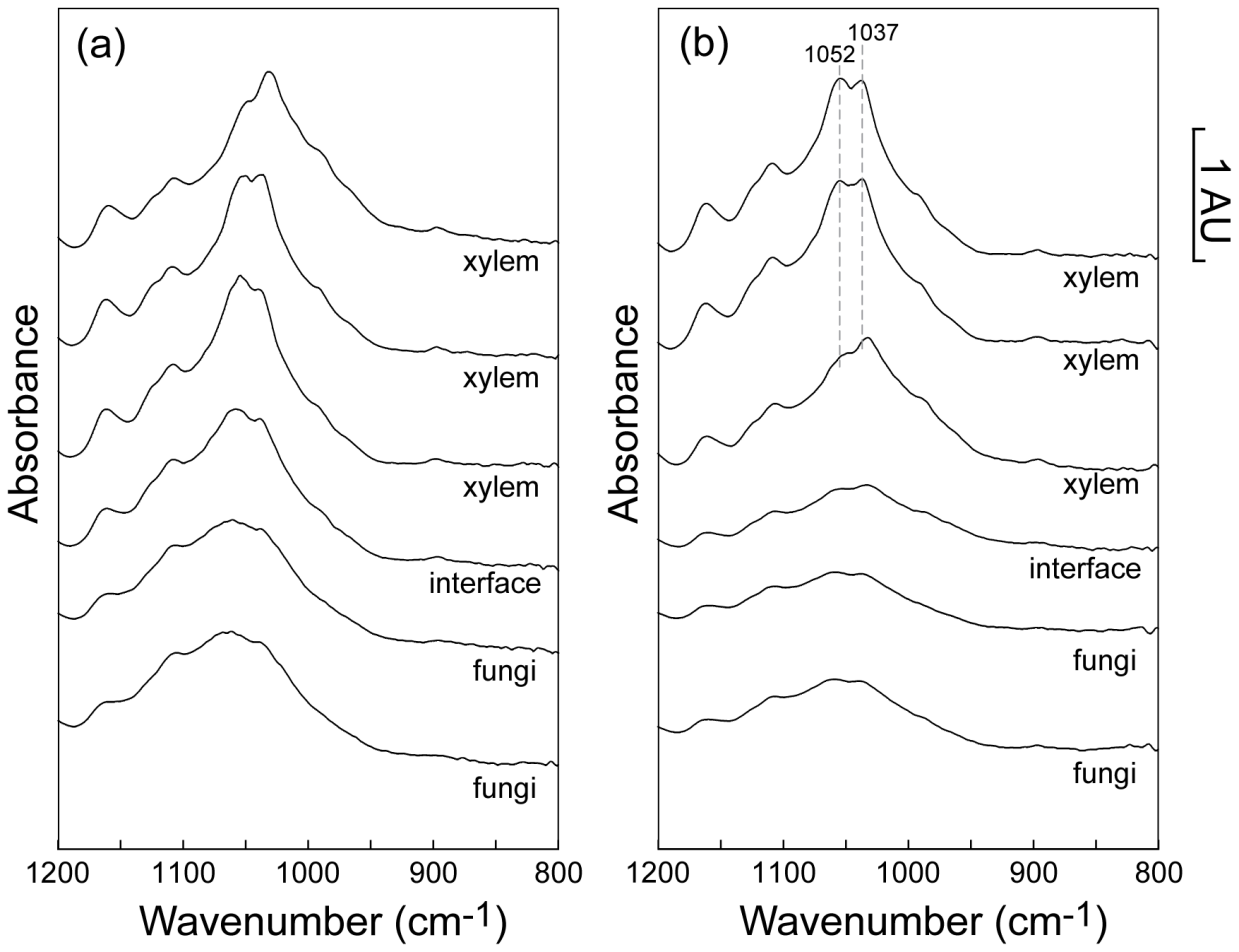
Example line scan spectra for the carbohydrate region across the fungal tissue-xylem interface. (1200 cm^−1^–800 cm^−1^) for: (**a**) a terrestrially aged *E. camaldulensis* leaf (**0**) and (**b**) an *E. camaldulensis* leaf after 15 days aquatic decomposition (**15**). Same pixel points as Figure 4.

**Figure 8:**
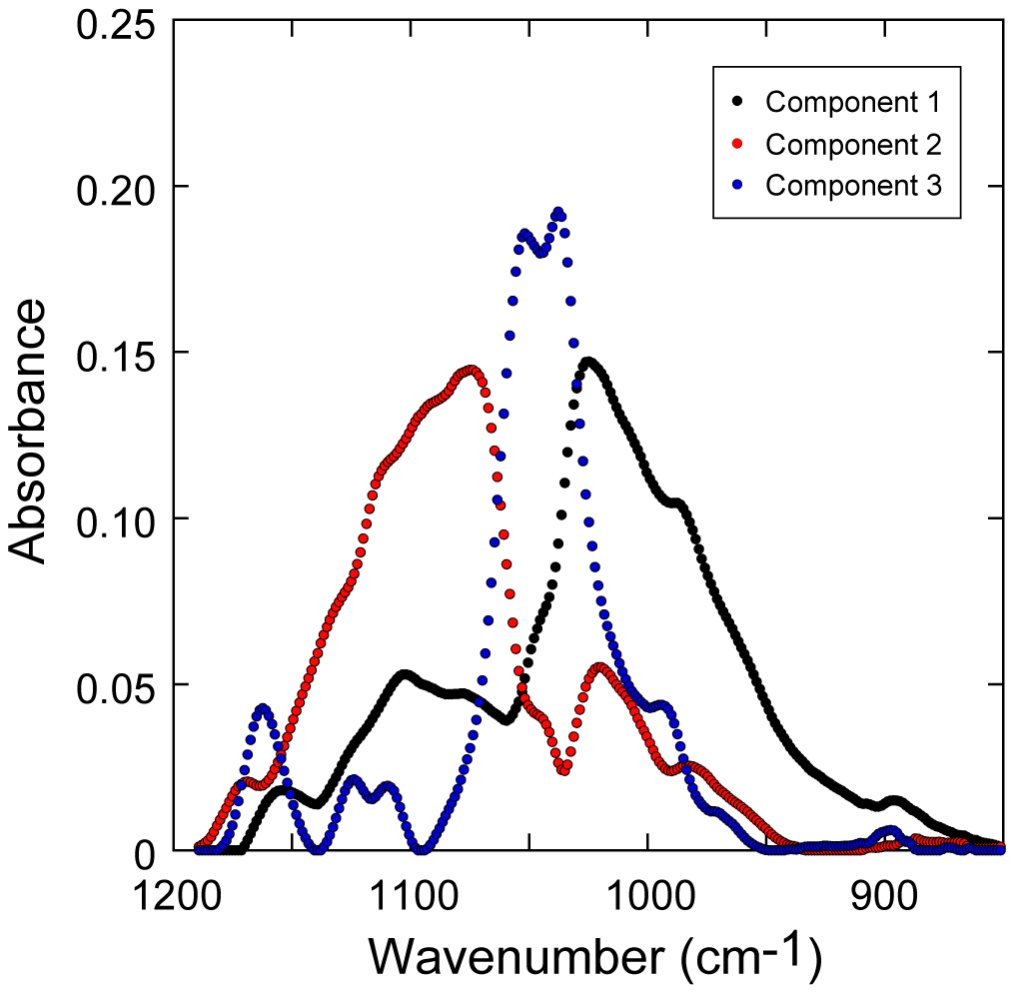
Component spectra for the carbohydrate region. (wavenumber range: 1190 cm^−1^–850 cm^−1^) obtained from multi-variate curve resolution (MCR) fitting of multiple pixel blocks (**0**, **15** & **107** – co-added) across the fungal tissue-xylem boundary. Spectral features of components described in main text.

An example of the spectral changes that occur across the fungal tissue-xylem interface in the wavenumber range 1190 cm^−1^–850 cm^−1^ are shown in Figure 7: part (a) shows spectra for the terrestrially aged leaf (**0**) and part (b) shows spectra after 15 days aquatic decomposition (**15**); same pixel points as shown in Figure 4. The data show the clear transition from the high carbohydrate content xylem to fungal tissue. Of particular importance are the prominent cellulose bands in unmodified xylem at 1052 cm^−1^ and 1037 cm^−1^ (marked). These peaks are significantly attenuated in the **15** sample towards the xylem-fungi interface; this attenuation trend is not observed in the **0** sample. Experimental spectra in this wavenumber range for the co-added pixel blocks were well described in MCR fitting by 3 spectral components (one outlier), shown in Figure 8. The three component fit of the carbohydrate region can be understood as follows. Components 1 and 2 describe the asymmetry in the carbohydrate band; component 1 represents carbohydrate bands at lower (spectroscopic) energy and component 2 represents carbohydrate bands at higher energy. Component 3 represents cellulose features with strong bands at 1052 cm^−1^ and 1037 cm^−1^ as well as weaker bands at 1160 cm^−1^ and 1120 cm^−1^ [27].

Using the same approach as that described for the carbonyl region, we have plotted the (fractional) contribution of cellulose (component 3) to the carbohydrate band against distance from the fungal tissue-xylem boundary, combining and averaging fractional contributions at common distances from this boundary. The cellulose line scan data is shown in Figure 6(b) for **0**, **15** and **107**. When compared to **0**, in both **15** and **107** the xylem immediately adjacent to fungal tissue is relatively depleted in cellulose, consistent with the selective removal of this bio-molecule from the xylem. Combined with the observed degradation of lignin in this same region, the spectral data are consistent with the fungal-mediated oxidative decomposition of lignin for the purpose of accessing the carbohydrate content of the xylem.

### Ecological significance

The high (spatial) resolution data obtained at the interface between fungal containing material and leaf lignin has shown that lignin oxidation and depletion of structural carbohydrates are spatially correlated and occur in close proximity to the fungal material. The data also suggest that lignin oxidation and cellulose depletion increase with increasing time of aquatic decomposition. While such a trend is likely, further verification will be required using replicated time series samples. We believe that this is the first direct evidence, obtained under natural ecological conditions, that supports a generally held view that aquatic fungi access lignin-bound carbohydrates [1], [6], [10]. Further, the results of this study suggest that the fungal degradation of xylem operates over relatively short length scales (5–15 µm) compared to the scale of the available lignified tissue in these leaves; a similar conclusion has been made for the penetration of lignin peroxidase into wood substrates [28]. It is likely that mechanical breakdown of vascular tissue through the combined actions of attrition and macroinvertebrate feeding activity is an important step in accessing the remaining carbohydrate contained in leaf xylem. In this study, leaves were contained in mesh bags and thus largely protected from mechanical breakdown.

We believe that the activity of aquatic fungi in degrading lignin may be an important step in the formation of a recalcitrant organic carbon pool in wetlands and an important link between carbon sequestration and aquatic food webs. The decomposition of leaf lignin by aquatic fungi partitions this carbon into an oxidized lignin fraction depleted in carbohydrate, and new fungal biomass that is likely more bioavailable than the original resource. This process may also be an important step in the formation of dissolved organic carbon (DOC) in aquatic systems. Lignin is a precursor of humic acid in aquatic systems [29]; relative to lignin, humic acid is more oxidized with higher abundance of carboxylic, phenolic and carbonyl (aldehyde and ketone) groups [30]. As demonstrated in this work, the exo-enzyme activity of aquatic fungi oxidizes and de-polymerises adjacent lignin, increasing the aldehyde and ketone content. These chemical transformations of lignin are likely to contribute to the formation of macromolecules of sufficient solubility that can enter the water column as DOC.

Allochthonous (externally derived) inputs of leaf material are an important source of carbon in aquatic ecosystems [31]. Terrestrial aging can remove most of the readily biodegradable material, yielding a substrate that is predominantly composed of leaf cuticle, lignified vascular tissue, aromatic materials of plant origin and fungal tissue. For macroinvertebrate feeder groups part of the direct nutritional value of the leaf is presumably the fungal tissue (both around the vascular tissue and in the mesophyll space), with perhaps a minor contribution from lignin bound carbohydrates mobilised by fungal activity. Further attrition of the leaf substrate within the gut may potentially increase the availability of both resources to the consumer organism.

## Experimental Methods

### Field sites and background

Fresh leaves were collected from a mature River Red Gum (*E. camaldulensis*) tree at Kiewa River Parklands, Killara, Victoria, Australia (−36.140093S, 146.955185E, 152 m elevation). Permission to collect leaves was by approval from Parklands Albury-Wodonga, the organisation responsible for the management of this site (formal permit not required; *E. camaldulensis* is not an endangered species). Multiple leaf discs (9 mm in diameter) were cut and preserved in 4% formaldehyde. These disks were used for standard histological examination as well as infrared (IR) spectroscopic mapping; labelled as “**Fr**.”.

Terrestrially aged leaves were collected from leaf litter on the floodplain of the Ovens River Nature Conservation Reserve, Peechelba East, Victoria, Australia (−36°9′44.72″S, 146°14′16.49″E; 137 m elevation; collection permit from Department of Sustainability and Environment (DSE, Victoria, Australia); Permit No. 10004708) and dried at 25°C for a period of 4 weeks in a dehydration oven. Leaf discs (9 mm in diameter) were cut from a sub-set of dried leaves and preserved in 4% formaldehyde. These disks were used for standard histological examination as well as IR spectroscopic mapping; labelled as “**0**”. The remaining dried leaves were used in the study of aquatic decomposition; these leaves were placed in “leaf bags” made from 500 µm heavy duty nylon mesh (to limit attack by aquatic fauna) and submerged at a depth of 20–30 cm in a wetland at Kiewa River Parklands, Killara (Figure S6). Permission to conduct the field study at this site was by approval from Parklands Albury-Wodonga (formal permit not required). Leaf packs (3 replicates) were removed from the wetland after time intervals of 2, 15, 30, 60 & 107 days. After transport to the laboratory (on ice) the leaves were gently rinsed under a stream of deionised water and leaf disks collected, as described for fresh and terrestrially aged leaves (above); these leaf samples are labelled according to the time sub-merged in the wetland: “**2**”, “**15**”, “**30**” ”, “**60**” & “**107**”; **2**, **30** and **60** were not studied using infrared microspectroscopy.

In parallel with histological and IR examination, leaf tissue samples were also collected for analysis of ergosterol (a fungal biomass proxy [15]), nitrogen content, hyphal biovolume, fungal community structure and leaf mass loss. These results will be reported elsewhere as part of a larger study comparing leaf decomposition rates in wetlands and rivers, however for the purpose of background information the fungal biomass results (determined from ergosterol analysis) are shown in Figure S7. A maximum fungal biomass is observed in the 15–30 day period. In this paper we focus particularly on the histology and IR characteristics of the following leaf samples: **Fr.** (fresh leaf; no fungal decomposition), **0** (terrestrially aged dry leaf; prior to aquatic decomposition), **15** (15 days aquatic decomposition; maximum fungal biomass) and **107** (107 days aquatic decomposition; near complete decomposition), representing the extreme states in the leaf decomposition process.

### Histological methods

Leaf disks were dehydrated in a series of alcohol baths. This was followed by clearing in xylene, paraffin infiltration and embedding in a paraffin block prior to sectioning on a rotary microtome, as detailed in Heraud *et al.* [17]. Transverse 8 µm sections of leaf tissue were mounted onto poly-L-lysine coated CaF_2_ slides (0.5 mm×25 mm; Crystan, UK). Paraffin was removed from the mounted sections in a xylene bath (3×) immediately prior to infrared analysis. Adjacent 6 µm transverse sections were prepared on optical slides for histological examination. These sections were stained with periodic acid-Schiff (PAS) reagent, which binds to glycogen and carbohydrates [32] and lacto-phenol cotton blue (LPCB) which stains fungal chitin [33].

### SEM sample preparation

Terrestrially aged leaf samples were cut obliquely along the centre vein and coated with Pt (25 mA; 15 kV; 50 secs) prior to imaging. Aquatically decomposed leaves (stored in 4% formaldehyde) were dehydrated in ethanol and then conditioned in hexamethyldisilazane (HMDS). After drying, the leaf sample was dissected to reveal the outer layer of the xylem, and then coated with Pt (25 mA; 15 kV; 50 secs). All SEM imaging was conducted using a JEOL JEM-6340; imaging conditions shown in micrographs.

### FPA and synchrotron IR mapping

All infrared (IR) maps were acquired in transmission mode, with the majority of data collected from the mid-vein and the adjacent mesophyll. Broad scale IR images of leaf sections were collected using the 15× objective of an Hyperion 3000 infrared microscope (Bruker Optik GmbH, Ettlingen, Germany) equipped with a focal plane array (FPA) rapid scan imaging system coupled to a Vertex 70 FT-IR spectrometer (Bruker Optik GmbH, Ettlingen, Germany). Opus 6.5 software (Bruker Optik GmbH, Ettlingen, Germany) was used to both control the instrument and process the spectra into chemical bond maps. Apodization was performed using Blackman-Harris 3-term function. FPA maps consisted of multiple ‘blocks’, each containing 1024 data points (32×32) and corresponding to a leaf area of 0.3 mm^2^.

Higher resolution synchrotron IR maps of leaf sections were acquired using the 36× objective of a Hyperion 2000 infrared microscope (Bruker Optics GmbH, Ettlingen, Germany) with a jacketed stage controlled to less than 70% humidity. The stage was motorized to enable point by point raster scanning of the sample. The microscope was coupled to a Vertex 80V IR spectrometer with a photovoltaic liquid nitrogen cooled mercury-cadmium-telluride (MCT) detector system (Bruker Optik GmbH, Ettlingen, Germany) and connected to the infrared light source at the Australian Synchrotron (Clayton, Australia). The system was controlled using Bruker Opus 6.5 software. The microscope's transparent knife-edge aperture was set to 5 µm; all images shown in this work were mapped at 5 µm step size. Background spectra (CaF_2_ slide) were collected after every 10–12 pixel points. Apodization was performed using the Happ-Genzel function.

For both FPA and synchrotron IR maps, data were collected in the spectral region of 3500 to 800 cm^−1^ at a resolution of 4 cm^−1^ with 64 scans co-added.

### Data processing and image analysis

The assignment of spectral bands observed in IR maps of *E. camaldulensis* leaf tissue sections are based primarily on previous work on *E. botryoides* leaves [17], with additional specific information on the IR spectra of fungals chitins [34], carbohydrates [27], lignin [24], [25] and eucalypt polyphenols [35]. More general features of IR spectra (H-bonding and conjugation effects) are taken from a standard IR textbook [21]. The IR absorption bands of particular interest in this work (1800–900 cm^−1^ region) are shown in Table 1, organised according to bio-molecule type.

Opus 6.5 software (Bruker Optik GmbH, Ettlingen, Germany) was used to analyse IR spectral maps. For all maps a standard set of wavenumber ranges were integrated. These ranges were: **(i)** 1705 cm^−1^–1570 cm^−1^, **(ii)** 1530 cm^−1^–1480 cm^−1^, **(iii)** 1260 cm^−1^–1210 cm^−1^), and **(iv)** 1180 cm^−1^–950 cm^−1^. The IR absorption bands mapped by these wavenumber ranges are listed in Table 2. The integration method applied was one whereby a line is drawn between the two wavenumber limits and the area of the curve above that line integrated. In the integrated x–y maps generated, the absorbance (z) in each wavenumber range is represented by a colour scale; in all maps the minimum absorbance has been forced to zero and maximum absorbance set at a consistent value for each wavenumber range.

The interface between fungal tissue and lignified leaf tissue (xylem) was analysed in greater detail using multivariate statistics. Selected pixel blocks across the fungal tissue-xylem interface were extracted from the S-FTIR data files with Opus 6.5, using bright field images to determine the fungal tissue-xylem boundary position. These blocks were 6 (or 5)×12 pixels, providing 12 pseudo-replicate line scans of 6 (or 5) pixels, and were extracted for terrestrially aged (**0**), 15 days aquatic decomposition (**15**) and 107 days aquatic decomposition (**107**) samples. Pixel blocks for **0**, **15** and **107** were co-added to form a single data file (198 spectra). These spectra were analysed by multi-variate curve resolution (MCR) techniques using Unscrambler 10.1 (CAMO Software AS, Oslo¸ Norway). A generalised approach to fitting was followed, including: (i) selection of the wavelength range (1800 cm^−1^–1480 cm^−1^ or 1190 cm^−1^–850 cm^−1^), (ii) baseline correction, (iii) MCR fitting using pure component initial guesses (1800 cm^−1^–1480 cm^−1^ only), and (iv) removal of outliers and re-fitting. The MCR fitting constraints were non-negative concentrations and non-negative spectra. The MCR algorithm in Unscrambler 10.1 uses an optimisation algorithm that minimizes the total residual across all wavelengths (j) and all spectra (i) as shown by equation 1, where ‘x’ is the experimental absorbance and ‘
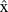
’ is the calculated absorbance.
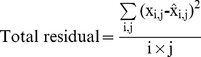
(1)


Goodness of fit is assessed through individual spectral residuals, calculated using equation 2.
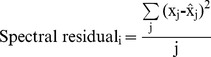
(2)


## Supporting Information

Figure S1 **SEM image of fungal material (F) both outside phloem fibres (PF) and between xylem (X) and PF for a terrestrially aged** ***Eucalyptus camaldulensis*** **leaf.**
(TIF)

Figure S2 **SEM image of fungal material (F) between xylem (X) and phloem fibres (PF) for a** ***Eucalyptus camaldulensis*** **leaf after 15 days aquatic decomposition.**
(TIF)

Figure S3 **Terrestrially aged leaf (0).** (**a**) bright field image, raster grid (red), selected pixel block for MCR analysis (yellow) and fungal tissue (F) – xylem (X) boundary position (yellow dots); (**b**) & (**c**) averaged scatter data (fractional contributions) across the fungal tissue-xylem boundary, aligned at the interface (zero position) with negative numbers indicating fungal tissue and positive numbers indicating xylem: Part (b) shows fractional contributions of component 2 (lignin) and components 1+3 (fungal tissue and aromatic materials) and part (c) shows fractional contribution of component 4 (‘oxidized lignin’) to experimental spectra. Error bars are 2×standard error.
(TIF)

Figure S4 **15 day aquatic decomposition leaf (15).** (**a**) bright field image, raster grid (red), selected pixel block for MCR analysis (yellow) and fungal tissue (F) – xylem (X) boundary position (yellow dots); (**b**) & (**c**) averaged scatter data (fractional contributions) across the fungal tissue-xylem boundary, aligned at the interface (zero position) with negative numbers indicating fungal tissue and positive numbers indicating xylem: Part (b) shows fractional contributions of component 2 (lignin) and components 1+3 (fungal tissue and aromatic materials) and part (c) shows fractional contribution of component 4 (‘oxidized lignin’) to experimental spectra. Also shown in (c) are the equivalent data for the terrestrially aged (**0**) leaf (from Figure S3). Error bars are 2×standard error.
(TIF)

Figure S5 **107 day aquatic decomposition leaf (107).** (**a**) bright field image, raster grid (red), selected pixel block for MCR analysis (yellow) and fungal tissue (F) – xylem (X) boundary position (yellow dots); (**b**) & (**c**) averaged scatter data (fractional contributions) across the fungal tissue-xylem boundary, aligned at the interface (zero position) with negative numbers indicating fungal tissue and positive numbers indicating xylem: Part (b) shows fractional contributions of component 2 (lignin) and components 1+3 (fungal tissue and aromatic materials) and part (c) shows fractional contribution of component 4 (‘oxidized lignin’) to experimental spectra. Also shown in (c) are the equivalent data for the terrestrially aged (**0**) leaf (from Figure S3). Error bars are 2×standard error.
(TIF)

Figure S6 **Air-dried terrestrially aged leaves were: (a) sown into 500 µm mesh litter bags and (b) submerged in the floodplain wetland at a depth of 20–30 cm.** The wetland was an ox-bow lake on the floodplain of the Kiewa River (**c**), at Killara in north-eastern Victoria, Australia, with riparian vegetation dominated by *Eucalyptus camaldulensis*.
(TIF)

Figure S7 **Fungal biomass in** ***E. camaldulensis*** **leaves (milligrams fungal tissue per gram leaf, dry weight) during aquatic conditioning, showing maximum fungal biomass at 15 days.** Error bars are 1× standard error.
(TIF)
