# Supplementary figures and images for: High Spatial Resolution Infrared Micro-Spectroscopy Reveals the Mechanism of Leaf Lignin Decomposition by Aquatic Fungi
Source: PLoS One. 2013 Apr 5;8(4):e60857. doi: 10.1371/journal.pone.0060857 (PMC3618115; doi:10.1371/journal.pone.0060857)

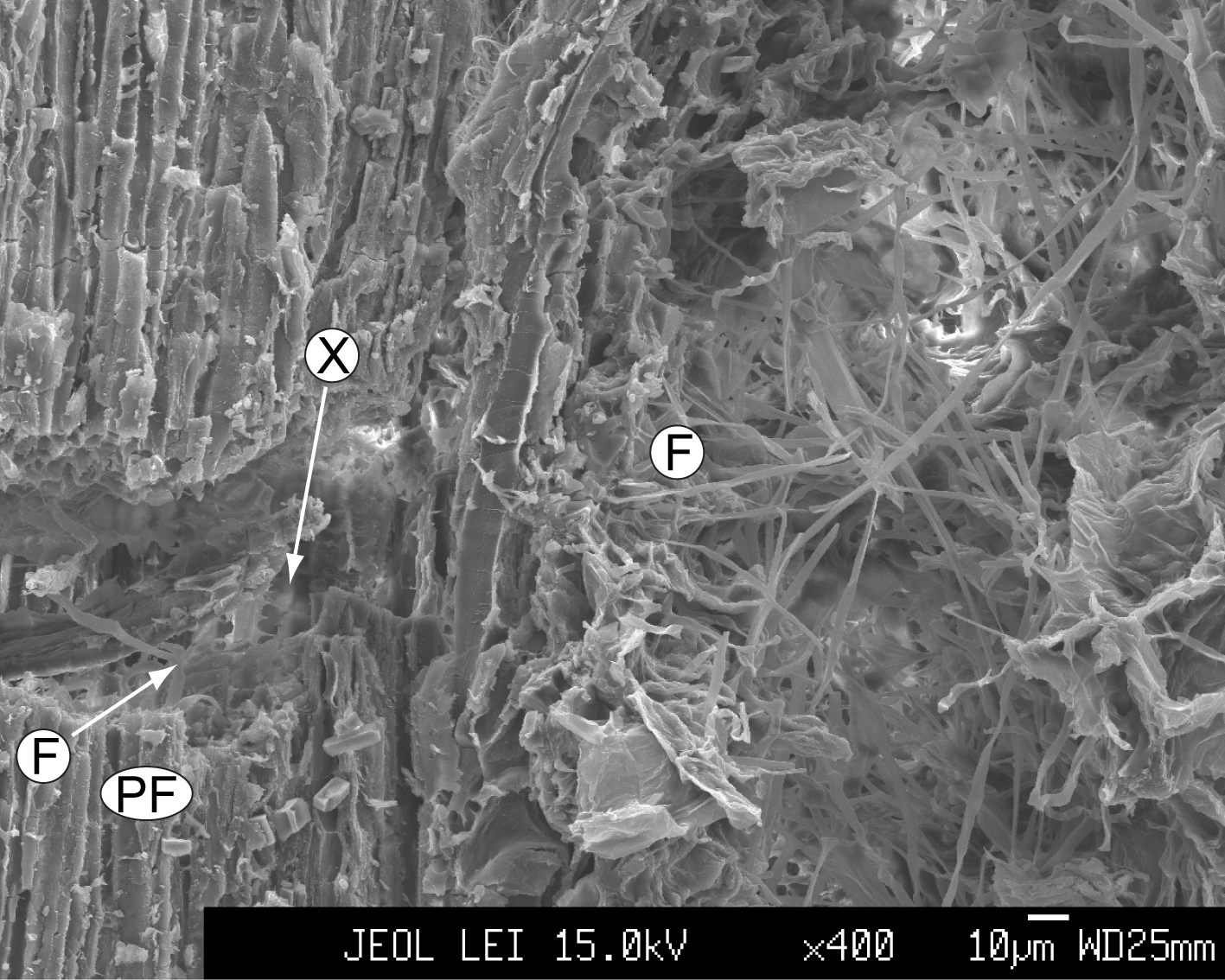

Supplement: Figure S1 — SEM image of fungal material (F) both outside phloem fibres (PF) and between xylem (X) and PF for a terrestrially aged Eucalyptus camaldulensis leaf. (TIF) [file pone.0060857.s001.tif]

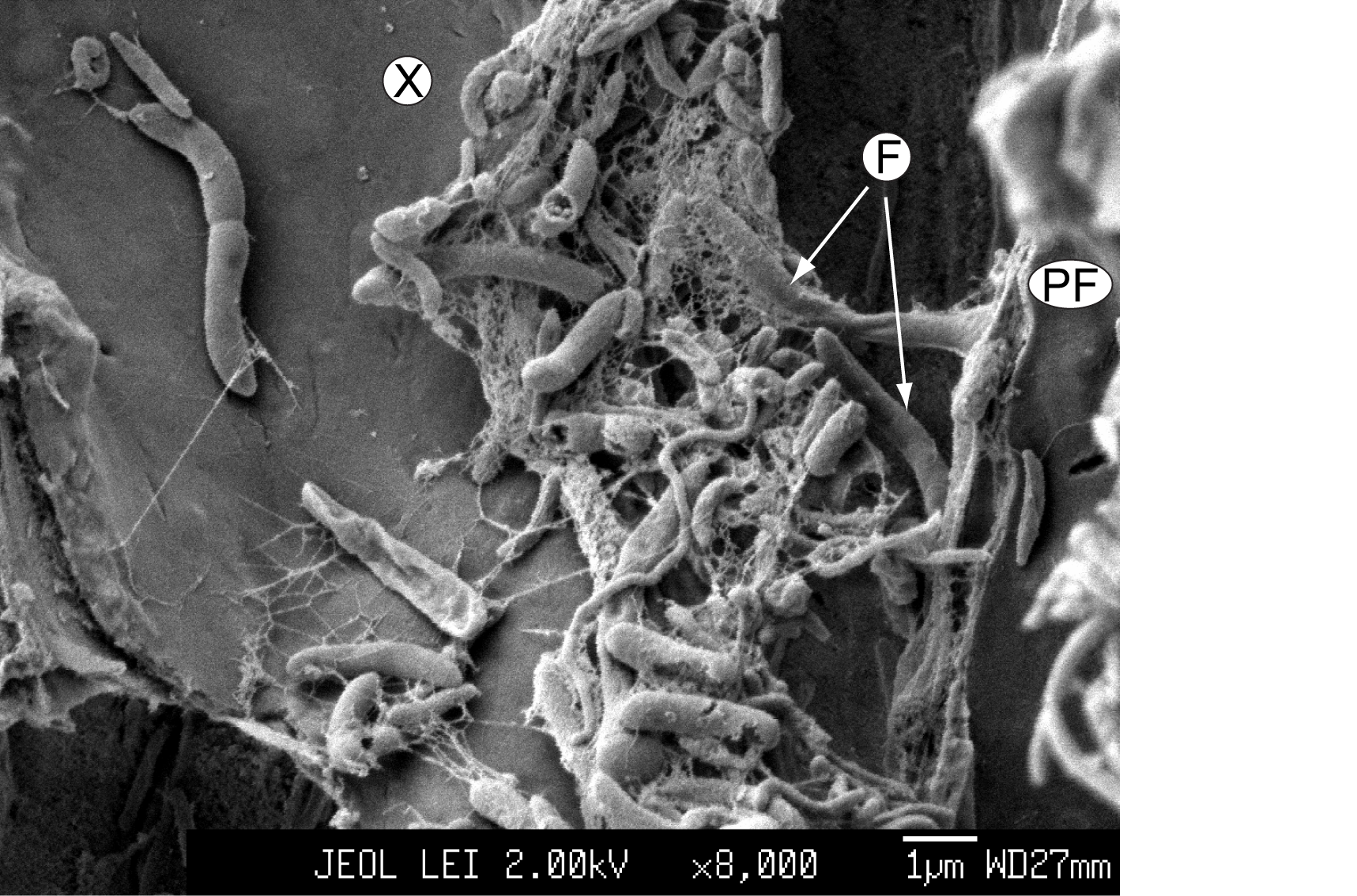

Supplement: Figure S2 — SEM image of fungal material (F) between xylem (X) and phloem fibres (PF) for a Eucalyptus camaldulensis leaf after 15 days aquatic decomposition. (TIF) [file pone.0060857.s002.tif]

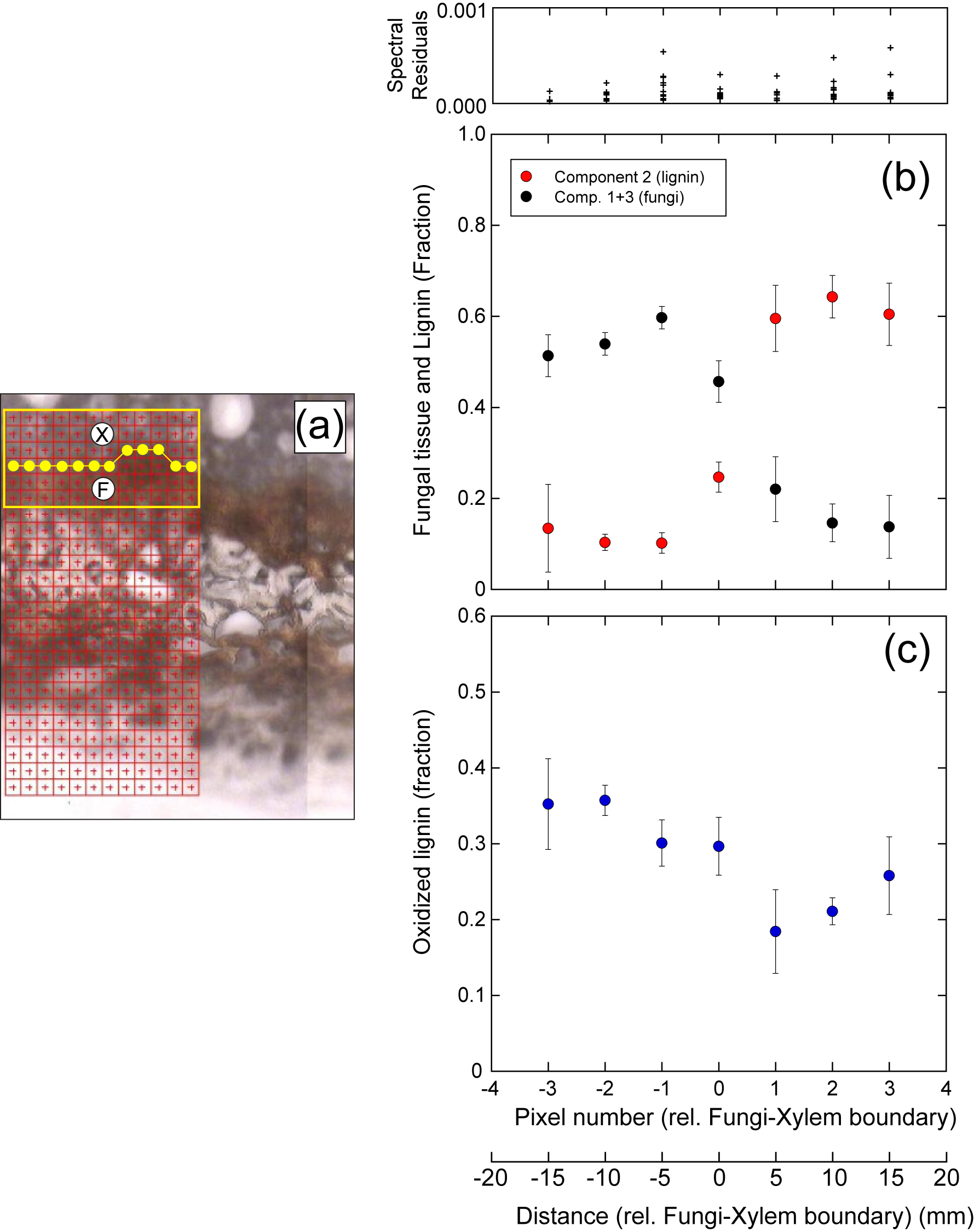

Supplement: Figure S3 — Terrestrially aged leaf (0). (a) bright field image, raster grid (red), selected pixel block for MCR analysis (yellow) and fungal tissue (F) – xylem (X) boundary position (yellow dots); (b) & (c) averaged scatter data (fractional contributions) across the fungal tissue-xylem boundary, aligned at the interface (zero position) with negative numbers indicating fungal tissue and positive numbers indicating xylem: Part (b) shows fractional contributions of component 2 (lignin) and components 1+3 (fungal tissue and aromatic materials) and part (c) shows fractional contribution of component 4 (‘oxidized lignin’) to experimental spectra. Error bars are 2×standard error. (TIF) [file pone.0060857.s003.tif]

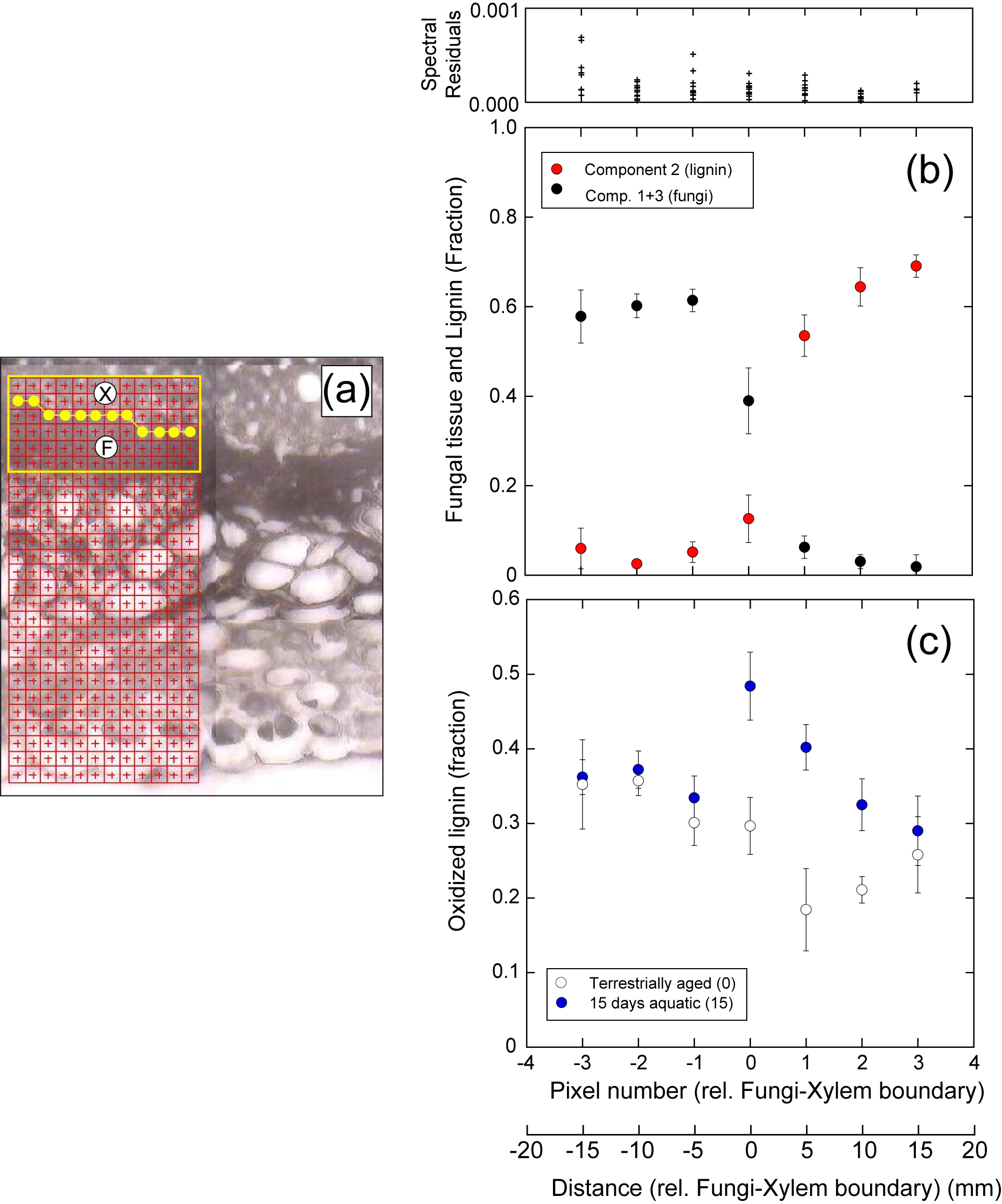

Supplement: Figure S4 — 15 day aquatic decomposition leaf (15). (a) bright field image, raster grid (red), selected pixel block for MCR analysis (yellow) and fungal tissue (F) – xylem (X) boundary position (yellow dots); (b) & (c) averaged scatter data (fractional contributions) across the fungal tissue-xylem boundary, aligned at the interface (zero position) with negative numbers indicating fungal tissue and positive numbers indicating xylem: Part (b) shows fractional contributions of component 2 (lignin) and components 1+3 (fungal tissue and aromatic materials) and part (c) shows fractional contribution of component 4 (‘oxidized lignin’) to experimental spectra. Also shown in (c) are the equivalent data for the terrestrially aged (0) leaf (from Figure S3). Error bars are 2×standard error. (TIF) [file pone.0060857.s004.tif]

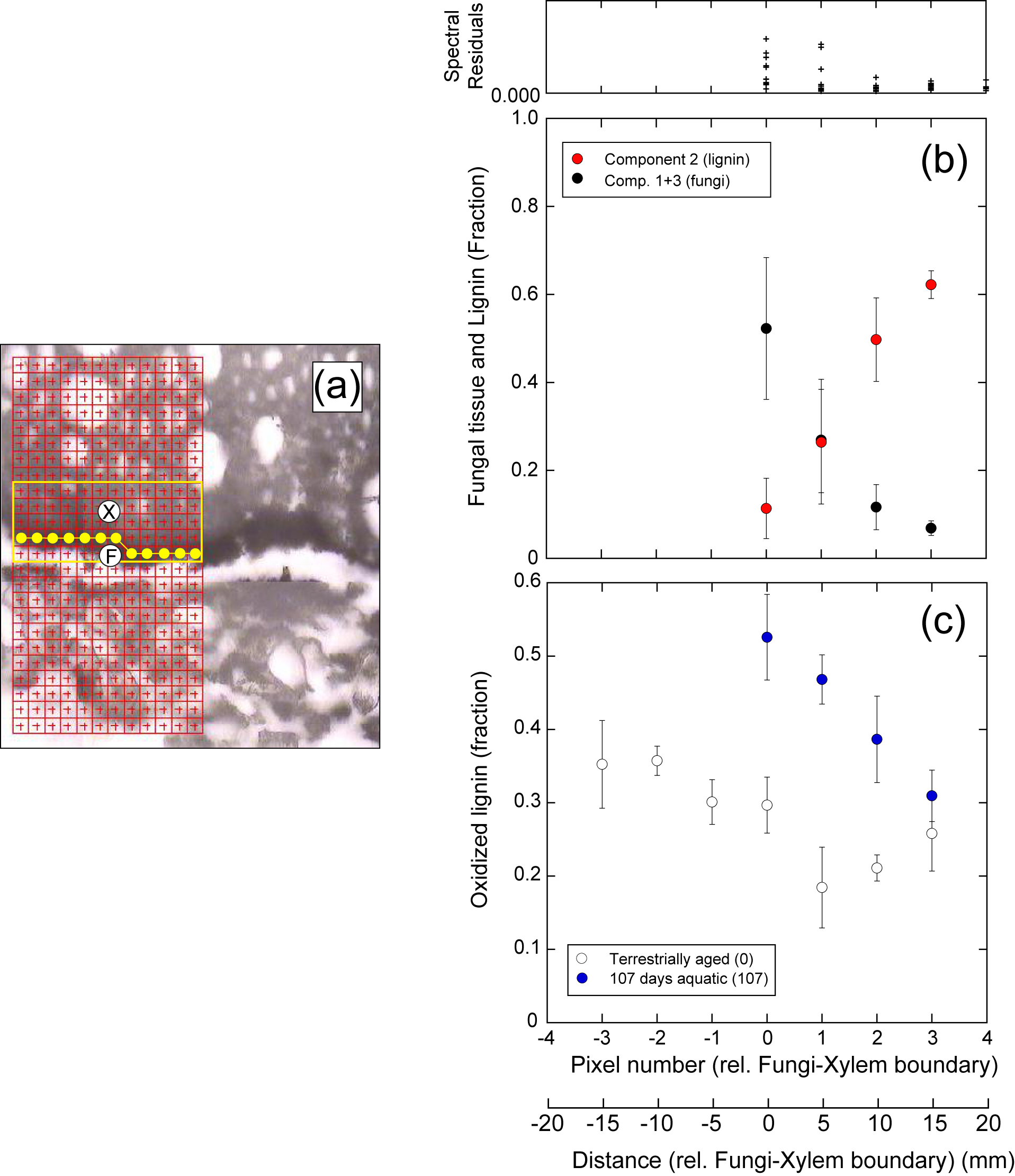

Supplement: Figure S5 — 107 day aquatic decomposition leaf (107). (a) bright field image, raster grid (red), selected pixel block for MCR analysis (yellow) and fungal tissue (F) – xylem (X) boundary position (yellow dots); (b) & (c) averaged scatter data (fractional contributions) across the fungal tissue-xylem boundary, aligned at the interface (zero position) with negative numbers indicating fungal tissue and positive numbers indicating xylem: Part (b) shows fractional contributions of component 2 (lignin) and components 1+3 (fungal tissue and aromatic materials) and part (c) shows fractional contribution of component 4 (‘oxidized lignin’) to experimental spectra. Also shown in (c) are the equivalent data for the terrestrially aged (0) leaf (from Figure S3). Error bars are 2×standard error. (TIF) [file pone.0060857.s005.tif]

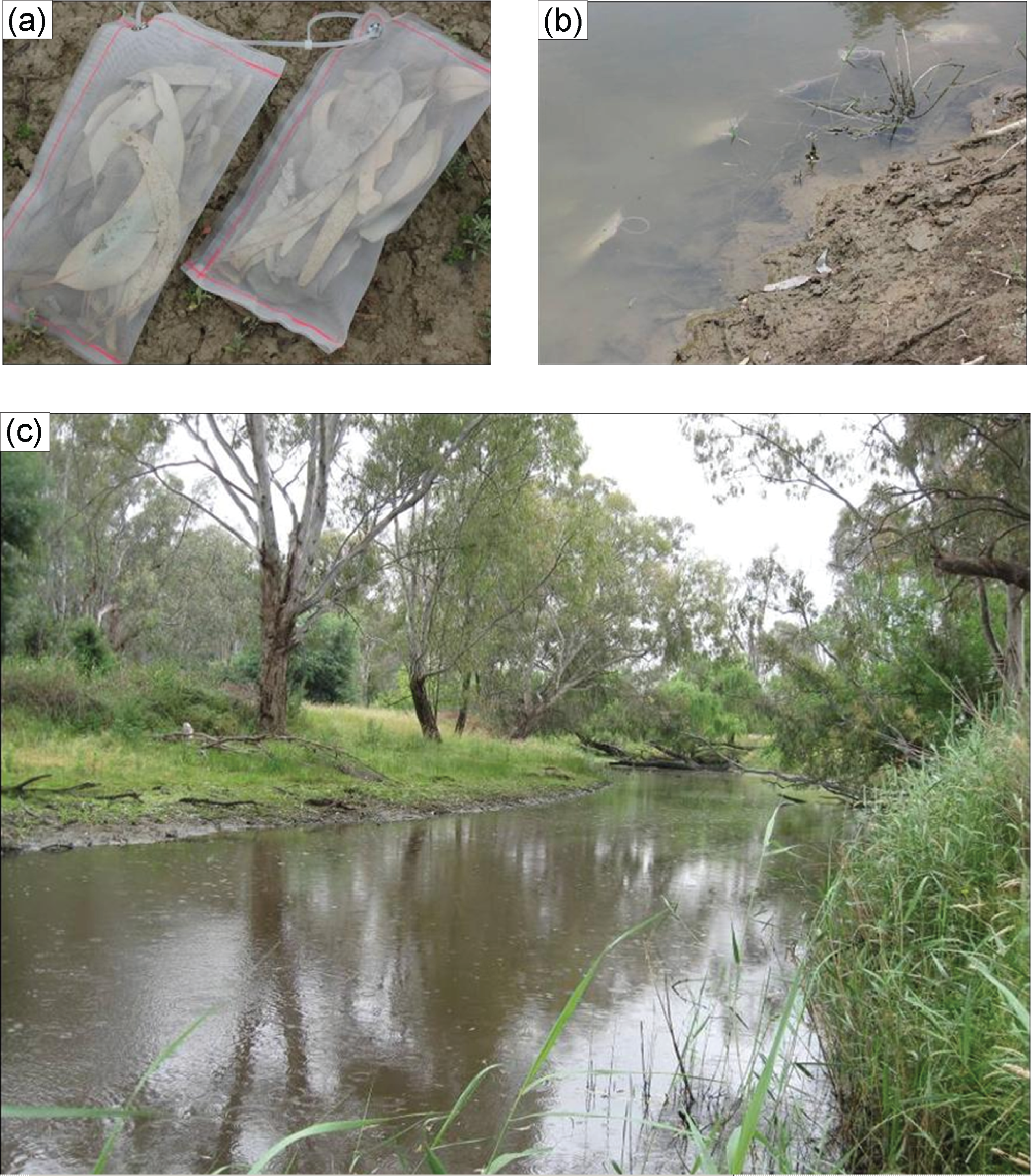

Supplement: Figure S6 — Air-dried terrestrially aged leaves were: (a) sown into 500 µm mesh litter bags and (b) submerged in the floodplain wetland at a depth of 20–30 cm. The wetland was an ox-bow lake on the floodplain of the Kiewa River (c), at Killara in north-eastern Victoria, Australia, with riparian vegetation dominated by Eucalyptus camaldulensis. (TIF) [file pone.0060857.s006.tif]

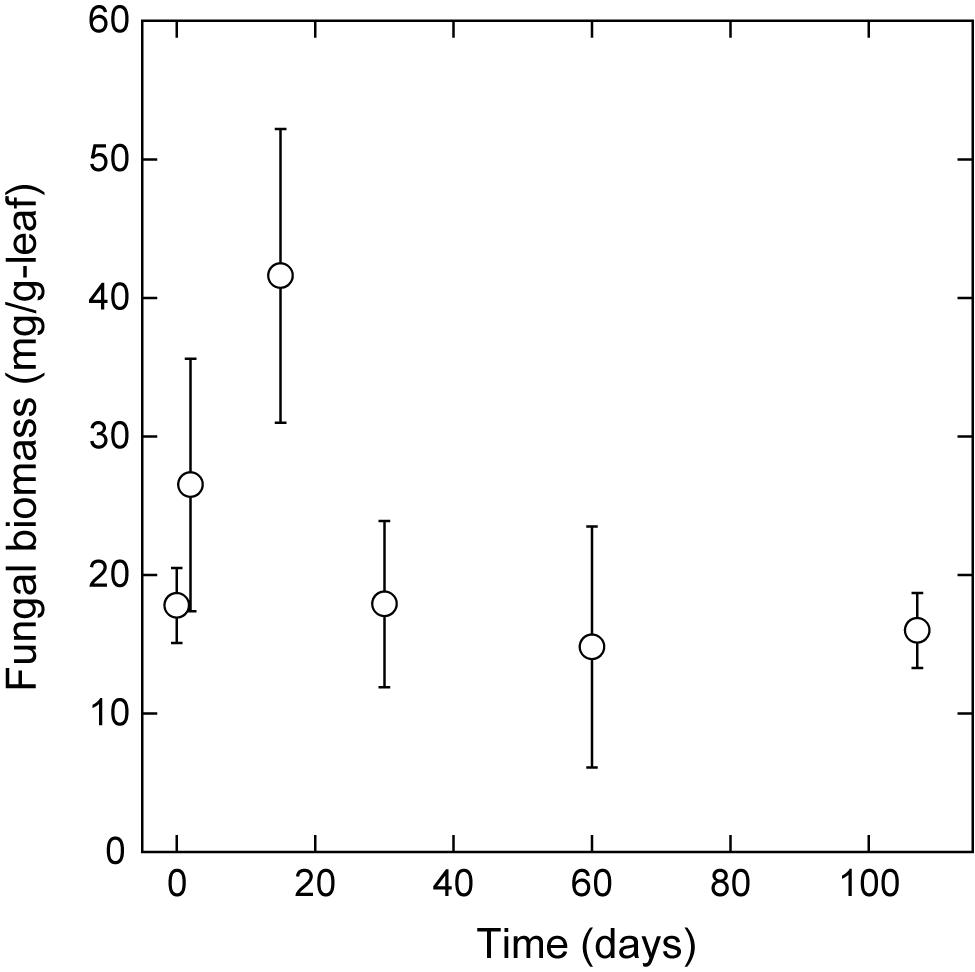

Supplement: Figure S7 — Fungal biomass in E. camaldulensis leaves (milligrams fungal tissue per gram leaf, dry weight) during aquatic conditioning, showing maximum fungal biomass at 15 days. Error bars are 1× standard error. (TIF) [file pone.0060857.s007.tif]
